# Automated Classification of Endometrial Pathologies Using Artificial Intelligence

**DOI:** 10.3390/biology15171490

**Published:** 2026-09-02

**Authors:** Vasileios Bais, Panagiotis Kokkas, Charikleia Skentou, Anastasia Vatopoulou, Minas Paschopoulos, Fani Gkrozou

**Affiliations:** 1 Department of Obstetrics and Gynaecology, School of Medicine, Faculty of Health Sciences, University of Ioannina, 45332 Ioannina, Greecefani.gkrozou@gmail.com (F.G.); 2 Department of Physics, University of Ioannina, 45110 Ioannina, Greece

**Keywords:** artificial intelligence, deep learning, hysteroscopy

## Abstract

Hysteroscopy provides direct visualization of the uterine cavity; however, diagnostic outcomes remain highly operator-dependent and subjective. This study aimed to develop an automated deep learning framework based on the ConvNeXt-XLarge architecture, in comparison with an EfficientNetV2 baseline, to objectively classify common uterine pathologies and thereby reduce diagnostic variability. A prospective dataset of 1262 histopathologically confirmed hysteroscopic images was analyzed using an 80:20 training to validation split and a two-stage transfer learning strategy. In addition to the primary multiclass framework, three binary classification models were developed to evaluate pairwise differentiation between the pathologies. The multiclass model achieved an accuracy of 80.5%, a precision of 82.3%, a weighted Area Under the Receiver Operating Characteristic Curve (AUC) of 94.8%, and a weighted average precision (AP) of 91.6%. In the binary classification tasks, model 1 (fibroids vs. functional polyps) achieved an accuracy of 96.7% and an AUC of 99.8%; model 2 (fibroids vs. hyperplastic polyps) achieved 87.2% accuracy and an AUC of 87.4%; and model 3 (functional vs. hyperplastic polyps) achieved 92.2% accuracy and an AUC of 99.0%. ConvNeXt-XLarge statistically significantly outperformed EfficientNetV2 in the multiclass task in terms of accuracy and AUC, and in model 2 in terms of accuracy, underscoring the added value of the deeper backbone for this hysteroscopic image classification. Overall, these findings demonstrate that deep learning frameworks can accurately automate the classification of hysteroscopic findings. By providing high-precision analysis, these models offer a robust tool to mitigate diagnostic subjectivity and optimize clinical decision-making.

## Simple Summary

Hysteroscopy is a procedure providing direct visualization of the uterine cavity; however, its diagnostic accuracy depends substantially on the physician’s subjective interpretation. To address this challenge, this study aimed to develop an automated computer-based system for the objective classification of common intrauterine conditions. By analyzing a dataset of 1262 clinical images, the model was trained to recognize distinct disease patterns. The framework achieved an overall accuracy of more than 80% in the multiclass classification task and up to 96.7% in the binary classification tasks involving specific pairs of pathologies. These findings demonstrate that advanced computing frameworks can successfully automate medical image interpretation in hysteroscopy and provide healthcare providers with a reliable and objective decision-support tool.

## 1. Introduction

Hysteroscopy is a minimally invasive endoscopic technique that enables direct visualization of the uterine cavity and is considered the gold standard for the diagnosis and management of intrauterine pathology [1]. It plays a critical role in the evaluation of common gynecological conditions, including abnormal uterine bleeding and infertility, by allowing direct assessment of the endometrial cavity [1]. Despite its clinical utility, hysteroscopic diagnosis relies on the operator’s assessment and clinical experience [2,3]. Endometrial polyps and submucosal fibroids have been reported as the most frequently encountered intrauterine lesions in hysteroscopy [4].

An endometrial polyp is an abnormal growth of the endometrium, consisting of glands, stroma, and blood vessels, that projects into the uterine cavity and may vary in size [5]. Morphologically, these lesions are classified as pedunculated when attached to the uterine wall by a pedicle, or sessile when they arise from a broad base [5]. Histologically, two distinct subtypes are recognized: functional and hyperplastic polyps. Functional polyps consist of responsive endometrium that undergoes normal cyclic changes, exhibiting a mid-secretory glandular pattern or a decidualized stroma in the late secretory phase. In contrast, hyperplastic polyps are characterized by actively proliferating glands within a hypercellular, endometrial-type stroma that fails to undergo physiological shedding during menstruation [5,6].

A uterine fibroid is a benign monoclonal tumor arising from the smooth muscle cells of the myometrium, characterized by an abundant accumulation of extracellular matrix [7]. Based on their anatomical location relative to the uterine wall, fibroids are categorized as submucosal, intramural, or subserosal [7]. This localization is formally standardized by the FIGO classification system, which assigns stages from type 0 to 8. Specifically, types 0, 1, and 2 represent submucosal lesions, ranging from intracavitary growths to those with varying degrees of intramural extension [8]. These categories are of particular clinical significance as they are directly accessible for definitive diagnosis via hysteroscopy and represent candidates for hysteroscopic myomectomy [9]. In contrast, types 3 through 7 describe intramural or subserosal tumors, whereas type 8 refers to ectopic locations, such as cervical or parasitic fibroids [8].

Artificial intelligence (AI) has become an integral component of modern medical image analysis, enabling fast, consistent, and highly accurate interpretation of complex visual data, supporting earlier diagnosis and more precise characterization of diverse pathologies [10,11]. Convolutional Neural Networks (CNNs) have emerged as the leading deep learning (DL) architecture for this purpose, designed to automatically learn spatial features from input data through a hierarchical process. This workflow begins with convolutional layers that employ mathematical kernels to extract essential patterns and subtle features that may be difficult for human observers to detect. These are followed by pooling layers that downsample the feature maps to maintain computational efficiency while preserving critical information. Finally, fully connected layers map these integrated features to specific output classes [12,13]. By leveraging large annotated datasets, CNN-based models can classify diseases, segment anatomical structures, and identify pathological biomarkers with performance that increasingly approaches, and in some cases surpasses, expert-level assessment [14,15]. Consequently, CNNs have been applied across a wide range of clinical domains, from diagnostic radiology [16] and pathology [17] to the broader surgical field [18], with notable advances recently reported in minimally invasive gynecologic surgery [19].

In particular, recent studies have explored the application of DL directly to hysteroscopic imaging. One study developed a DL-based clinical decision system to classify hysteroscopic images extracted from high-resolution videos into normal endometrium, endometrial polyps, and endometritis in in vitro fertilization (IVF) populations, reporting classification accuracies of 85% to 89% [20]. Another study introduced a DL model for differentiating atypical endometrial hyperplasia and endometrial cancer from benign lesions in hysteroscopic images, achieving high sensitivity and specificity and, in some settings, outperforming experienced endoscopists [21].

More recently, the landscape of computer vision has been transformed by the introduction of Vision Transformers (ViTs) [22,23]. Unlike CNNs, which rely on localized receptive fields, ViTs analyze the image by dividing it into a grid of smaller patches. By replacing traditional convolutional operations with self-attention mechanisms that capture global spatial relationships across the image, ViTs have achieved elite performance in large-scale image classification tasks. However, this improved performance comes at the cost of higher computational demands and the need for very large training datasets [22,23].

In response to this development, ConvNeXt-XLarge is a modern CNN developed by Meta researchers in 2022 to match or exceed ViTs in image classification tasks by strategically incorporating their most effective design principles. Specifically, it adopts larger kernels for broader visual context, layer normalization instead of traditional batch normalization for improved training stability, and the Gaussian Error Linear Unit (GELU) activation function instead of the Rectified Linear Unit (ReLU) for smoother and more sophisticated decision-making, all within a sparser overall structure that mimics the architectural simplicity of ViTs. In parallel, it retains highly efficient classic CNN features, utilizing skip connections to prevent information loss in deep networks, and depth-wise separable convolutions to smartly separate spatial learning from channel mixing by processing color channels individually to find shapes before merging them for efficient processing. By combining these ViT-inspired upgrades with foundational convolutional efficiency and training on the massive ImageNet-22K dataset, ConvNeXt-XLarge achieves exceptional accuracy on complex visual tasks while remaining faster and less computationally demanding than pure ViT models [24].

The aim of this study is to develop and evaluate an automated diagnostic framework for the classification of the most common uterine pathologies using high-resolution hysteroscopic images. Specifically, deep transfer learning has been applied and using the ConvNeXt-XLarge architecture, we are aiming to accurately recognize submucosal fibroids, functional polyps, and hyperplastic polyps. The study establishes both a primary multiclass prediction model and three binary classification models designed to evaluate all possible pairwise combinations between these three specific categories. Ultimately, this study aims to determine whether the ConvNeXt-XLarge multiclass and binary classifiers outperform corresponding models based on the simpler EfficientNetV2 architecture trained on the same hysteroscopic datasets, thereby quantifying the added value of the heavier ConvNeXt-XLarge backbone over a lightweight benchmark in this uterine-pathology classification setting.

## 2. Materials and Methods

The methodology of this study focused on the development of automated diagnostic frameworks for classifying uterine pathologies using deep transfer learning on high-resolution hysteroscopic imaging.

### 2.1. Dataset

The dataset used in this study was prospectively collected from hysteroscopy procedures performed at the University Hospital of Ioannina, Greece, a single-center tertiary institution, between December 2025 and June 2026. The final cohort comprised 1262 high-resolution hysteroscopic images representing three distinct pathologies. Particularly, 650 images of hyperplastic polyps, 439 of functional polyps and 173 of fibroids were included. In all cases, the diagnosis was confirmed via histopathological examination following surgical excision, ensuring a definitive ground truth for model training and evaluation. All images were resized to 384 × 384 pixels using bilinear interpolation to better preserve morphological details that are crucial for hysteroscopic diagnosis, deviating from the standard ImageNet resolution of 224 × 224 [25].

In addition to the multiclass framework, the dataset was also partitioned to support three distinct binary classification models, covering all possible combinations of the categories:

- Model 1: Fibroids vs. functional polyps
- Model 2: Fibroids vs. hyperplastic polyps
- Model 3: Functional polyps vs. hyperplastic polyps

All datasets were divided into training and validation sets using a standardized 80 to 20 percent split ratio. For the primary multiclass model, the dataset consisted of 1262 images, resulting in a training set of 1011 images and a validation set of 251 images. The same 80 to 20 percent split was applied to the data subsets used for the three binary models. Accordingly, model 1 utilized 612 total images partitioned into 491 for training and 121 for validation. Similarly, model 2 was trained on 823 total images split into 659 for training and 164 for validation, while model 3 employed 1089 total images distributed as 872 for training and 217 for validation. To ensure reliability and reproducibility, a fixed random seed was applied to guarantee consistent data shuffling and selection [26]. This standardization process ensured that the exact same validation images were utilized during both the convergence analysis and the final performance evaluation.

### 2.2. Data Augmentation

To improve model generalization model and prevent overfitting, a dual-stage augmentation pipeline was implemented to simulate the dynamic environment of clinical hysteroscopy.

During the training phase, images were subjected to random 30-degree rotations, horizontal flips, and 20% width and height shifts using the ImageDataGenerator of Keras 3.10.0, a high-level DL application programming interface built on top of TensorFlow that facilitates model training [27]. Simultaneously, integrated preprocessing layers added stochastic zoom (10%) and additional random flips. This forced the network to learn robust morphological features rather than memorizing static pixel patterns.

During the validation phase, the protocol was differentiated to ensure a realistic evaluation. While internal model layers as random zoom were automatically deactivated to maintain structural consistency, the geometric rotations and shifts via ImageDataGenerator remained active. This approach served as a real-world simulation, ensuring the network could maintain diagnostic accuracy despite the unpredictable camera angles and off-center framings typical of live hysteroscopic procedures.

### 2.3. Model Training and Fine-Tuning Strategy

The classification models were developed using the ConvNeXt-XLarge architecture, which was pre-trained on the ImageNet-22K dataset [24]. This dataset contains approximately 14.2 million images across more than 21,000 distinct categories, providing the network with the ability to recognize complex shapes and textures before any medical-specific training. For this study, the models were further trained and specialized using the hysteroscopic datasets described above. The system was implemented using the TensorFlow 2.16.1 deep learning framework in Python 3.9.25 on a workstation equipped with a dedicated NVIDIA RTX 3060 GPU with 12GB of VRAM (NVIDIA Corporation, Santa Clara, CA, USA).

The training process was executed in two distinct stages for both the multiclass and the binary classification models, although the number of epochs varied depending on the task:

#### 2.3.1. Multiclass Model Training Pipeline

- Phase 1 (Feature Extraction): Τhe base ConvNeXt-XLarge model was frozen to act as a fixed feature extractor. A custom classification head was trained for 50 epochs with an initial learning rate of 0.001. This relatively higher learning rate allowed the model to rapidly learn the distinctions between the three uterine pathologies in our dataset without altering the pre-trained weights of the backbone.
- Phase 2 (Selective Fine-Tuning): The base model was set to trainable, but most of the network remained frozen to preserve established features. Specifically, only the final 20 layers, consisting of the last functional blocks of the architecture, were unfrozen. Τhe learning rate was reduced by a factor of 100 to 0.00001, allowing the deepest parts of the CNN to adapt their weights specifically to the unique morphological characteristics and patterns of the hysteroscopic images. This phase was conducted for an additional 50 epochs.

#### 2.3.2. Binary Classification Models Training Pipeline

For each of the three binary classification systems, the same two-phase strategy was employed but optimized for a shorter duration. The initial feature extraction phase was conducted for 30 epochs, followed by 30 epochs of selective fine-tuning, resulting in a total training duration of 60 epochs for each binary model.

#### 2.3.3. Learning-Gap Analysis

To quantify training stability and potential overfitting, a learning-gap analysis was performed for all ConvNeXt-XLarge models. At each epoch, the difference between training accuracy and validation accuracy was calculated, together with the corresponding loss gap.

#### 2.3.4. Benchmark Architecture

In addition to the ConvNeXt-XLarge backbone, we trained a benchmark model based on the EfficientNetV2 architecture on the same hysteroscopic datasets across both the primary multiclass framework and all three binary classification tasks, using identical train-validation splits, augmentation settings, and optimization parameters. EfficientNetV2 is a lightweight, widely used CNN designed for parameter-efficient and fast training [28], and thus served as a practical baseline to quantify the added value of the heavier ConvNeXt-XLarge model in this high-resolution hysteroscopic classification setting, for both multiclass diagnosis and pairwise binary discrimination of uterine pathologies.

#### 2.3.5. Fine-Tuning Impact Analysis

Additional ConvNeXt-XLarge models were trained without selective fine-tuning, using only the initial feature-extraction phase (50 epochs for the multiclass task and 30 epochs for each binary task). Their performance was subsequently compared with the corresponding fine-tuned ConvNeXt-XLarge models.

### 2.4. Statistical Evaluation and Metrics

Statistical analysis was performed using a custom-built pipeline in Python 3.9.25, leveraging the Scikit-learn 1.6.1, NumPy 1.26.4, and Pandas 2.3.3 libraries for data manipulation and metric computation.

The evaluation methodology was tailored based on the classification task. For the multiclass model evaluation, to ensure a granular assessment across the three uterine pathologies, metrics were calculated for each category individually using a one-vs-rest (OvR) approach. Overall model performance was assessed using weighted-average metrics to account for class imbalance, ensuring that each pathology’s contribution to the final global scores was proportional to its sample size. In contrast, the three binary models were evaluated using standard binary configurations, in which one specific pathology was explicitly designated as the positive class and the alternative as the negative class. This straightforward dichotomy allowed performance metrics to be derived directly from true and false positive and negative assignments, eliminating the need for OvR approximations or multiclass weighting.

Performance for all models was evaluated based on standard statistical measures derived from True Positives (TP), True Negatives (TN), False Positives (FP), and False Negatives (FN):

- Accuracy: Reflects the proportion of correctly classified samples.

Accuracy = (TP + TN)/(TP + TN + FP + FN)

- Precision: Measures the reliability of positive diagnostic predictions.

Precision = TP/(TP + FP)

- Recall/Sensitivity: Assesses the ability of the model to identify all true positive cases.

Recall = TP/(TP + FN)

- Specificity: Εvaluates the capacity to correctly identify negative cases.

Specificity = TN/(TN + FP)

- F1-score: Provides a balanced measure that harmonizes precision and recall to ensure a robust assessment despite uneven class distributions [29].

F1-score= 2 × (precision × recall)/(precision + recall)

To evaluate the discriminative power and threshold-independent performance of the DL models, Receiver Operating Characteristic (ROC) analysis was conducted, and the Area Under the Curve (AUC) was computed. For the binary classification models, the standard Area Under the ROC Curve (ROC-AUC) was calculated. For the primary multiclass model, class-specific one-vs-rest (OvR) ROC curves were generated, together with macro-, micro-, and weighted-average ROC-AUC estimates. The macro-average AUC was calculated as the unweighted mean of the class-specific OvR AUC values, assigning equal importance to each diagnostic category. The micro-average AUC was calculated by pooling the OvR decisions across all samples and classes, whereas the weighted-average AUC was calculated by weighting the class-specific AUC values according to the number of samples in each class [30]. Additionally, Precision-Recall (PR) analysis was performed for the multiclass ConvNeXt-XLarge model. Class-specific PR curves were generated using a OvR formulation, and the corresponding Average Precision (AP) values were computed for fibroids, functional polyps, and hyperplastic polyps. In addition, a weighted AP summary was calculated to provide an overall measure of multiclass precision-recall performance [31].

Finally, 95% Confidence Intervals (CIs) were computed for all primary metrics. CIs for accuracy, precision, recall and specificity were derived using the normal approximation of the binomial distribution, as these metrics represent proportions of independent categorical successes. In contrast, the F1-score CIs were estimated via non-parametric bootstrapping with 1000 resampling iterations. For the ROC-AUC metrics, 95% CIs for the individual and binary curves were mathematically computed using the DeLong method to rigorously account for the variance of the Mann-Whitney statistic. The 95% CIs for the multiclass macro-, micro-, weighted-average ROC-AUC, and weighted average AP estimates were obtained using non-parametric bootstrap resampling with 1000 iterations. This approach provided complementary summaries of multiclass discrimination while accounting for the unequal prevalence of the diagnostic categories.

To statistically compare the ConvNeXt-XLarge backbone with the EfficientNetV2 benchmark, both models were evaluated on the same validation images using identical image ordering. Accuracy comparisons were performed using McNemar’s test for paired correct and incorrect predictions. This test was applied to the overall multiclass accuracy and separately to the accuracy of each binary classification model. For binary ROC-AUC values, both the comparison between ConvNeXt-XLarge and EfficientNetV2 and the internal comparison between fine-tuned and non-fine-tuned ConvNeXt-XLarge models were assessed using the paired DeLong test. The difference between multiclass weighted-average AUC values was evaluated using a paired non-parametric bootstrap procedure with 2000 resampling iterations to estimate the 95% confidence interval, and its statistical significance was tested with a paired permutation test based on 2000 permutations. A two-sided *p*-value below 0.05 was considered statistically significant.

### 2.5. Computational Benchmarking

Computational performance was evaluated for the multiclass and three binary ConvNeXt-XLarge models using the same input resolution of 384 × 384  and a batch size of one. Inference latency was measured on an NVIDIA GeForce RTX 3060 GPU with 12 GB of VRAM after 30 warm-up runs and over 100 repeated inference runs. Mean, median, standard deviation, 95th-percentile latency, and throughput were recorded. The benchmark measured model-only inference and excluded image acquisition and preprocessing time.

### 2.6. Occlusion-Sensitivity Analysis

To provide a qualitative indication of the image regions influencing the model’s decision, occlusion-sensitivity analysis was performed on a representative correctly classified fibroid image from the validation set. The input image was resized to 384 × 384 pixels, and non-overlapping 32 × 32-pixel patches were sequentially replaced with the mean RGB color of the image. The confidence of the predicted class was first recorded for the original, unmasked image and then recalculated after masking each individual patch. For each patch, its importance was quantified as the decrease in predicted-class confidence relative to the original unmasked image.

The resulting confidence-drop values were represented as a spatial occlusion-sensitivity map and superimposed on the original hysteroscopic image. Warmer colors indicate regions whose masking caused a greater decrease in the confidence of the predicted class and therefore had a stronger influence on the model’s decision. In contrast, darker regions indicate areas whose masking produced little or no decrease in the model output.

## 3. Results

### 3.1. Training, Fine-Tuning, and Learning-Gap Analysis of the Multiclass Model

**Figure 1:**
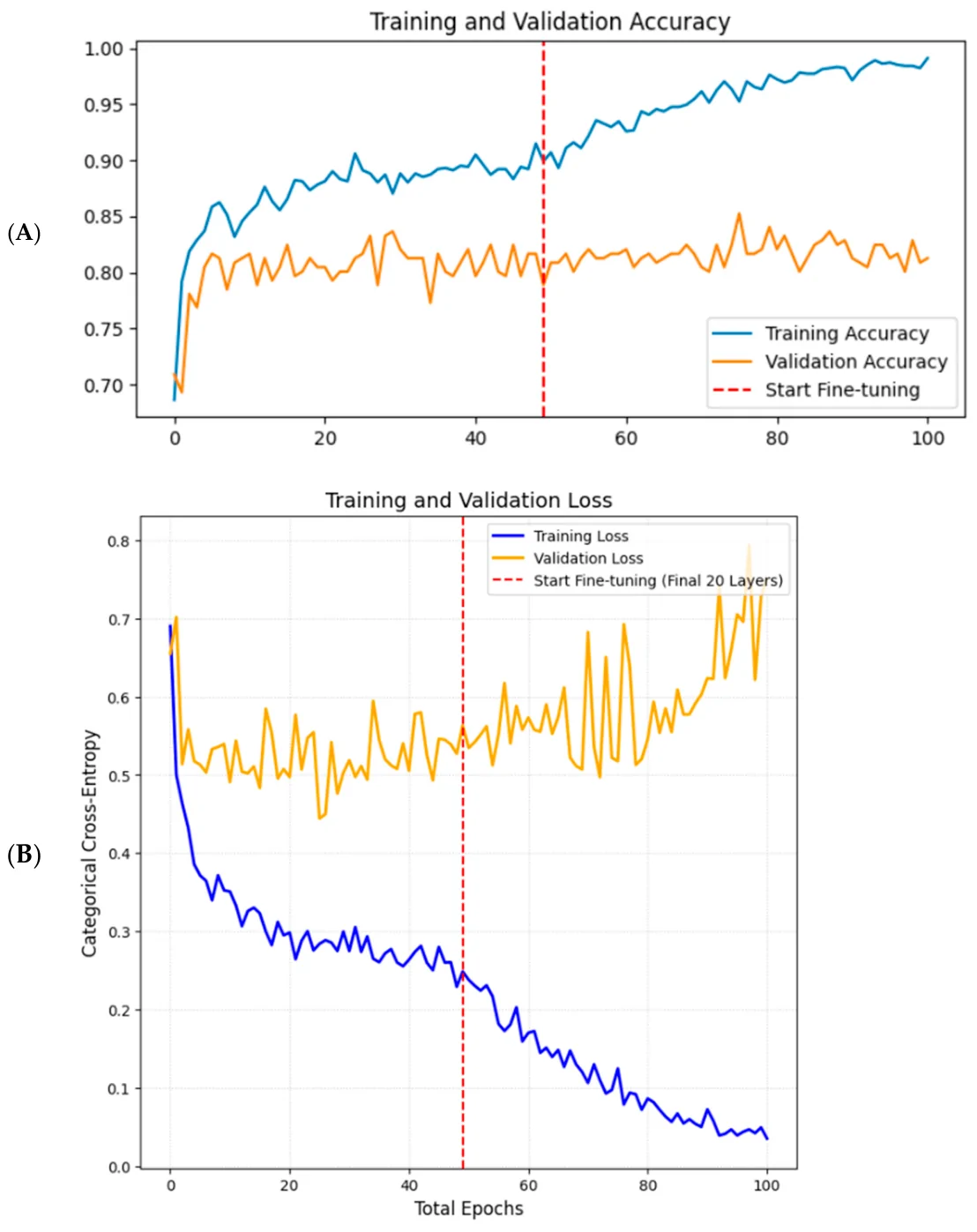
(**A**) Dual-phase learning curves for training and validation accuracy of multiclass prediction model. (**B**) Dual-phase learning curves for training and validation categorical cross-entropy loss of multiclass prediction model.

The ConvNeXt-XLarge multiclass model was trained for 100 epochs, comprising 50 epochs of feature extraction followed by 50 epochs of selective fine-tuning (Figure 1). To quantify overfitting, the learning gap was calculated at each epoch as the difference between training and validation accuracy, while the loss gap was calculated as validation loss minus training loss.

The learning-gap analysis demonstrated progressive overfitting during the later fine-tuning phase (Figure S1, Table S1). At the epoch of best validation accuracy, training accuracy was 96.4% and validation accuracy was 85.26%, corresponding to an accuracy gap of 11.14 percentage points. The maximum accuracy gap reached 18.45 percentage points at epoch 92. At the final epoch, training accuracy reached 99.48%, whereas validation accuracy was 81.27%, resulting in a final accuracy gap of 18.21 percentage points. Training loss was 0.0296 compared with a validation loss of 0.7497, corresponding to a loss gap of 0.7201. These findings indicate continued improvement in the training data but limited generalization to the validation data during the later fine-tuning phase.

### 3.2. Diagnostic Performance of the Multiclass Model

To thoroughly assess the clinical efficacy of the primary multiclass architecture, the validation set was evaluated using both global and class-specific metrics.

#### 3.2.1. Global and Categorical Diagnostic Performance

The model achieved a weighted-average accuracy of 80.5% (95% CI: 75.6–85.4%), a precision of 82.3% (95% CI: 77.6–87%), a recall of 80.5% (95% CI: 75.6–85.4%), and a specificity of 81.8% (95% CI: 77–86.6%). The harmonic balance between these metrics was reflected in an F1-score of 79.9% (95% CI: 74.7–84.8%) (Table 1). At the aggregate level, the model correctly identified 202 samples across all three categories, with 49 FP and 49 FN errors (Table S2A,B).

The classification performance for each uterine pathology, including the raw counts for TP, TN, FP, and FN, is presented in the categorical confusion matrix in Table S2. To further characterize diagnostic performance, class-specific performance metrics, including accuracy, precision, sensitivity, specificity, and F1-score, were calculated and are reported in Table 1. Distinct diagnostic profiles were observed across the three pathological categories.

Functional polyps demonstrated the highest diagnostic certainty, achieving a precision of 98.5% (95% CI: 95.5–100%) and a specificity of 99.4% (95% CI: 98.2–100.0%). The sensitivity for this class was 73.6% (95% CI: 64.3–82.8%), resulting in an F1-score of 84.2% (95% CI: 77–89.7%).

Hyperplastic polyps exhibited the highest sensitivity among all categories at 93.9% (95% CI: 89.7–98.0%), correctly identifying 122 out of 130 cases. However, this higher detection rate was accompanied by a relatively larger number of FP classifications, resulting in a lower specificity of 66.1% (95% CI: 57.7–74.6%) and a precision of 74.9% (95% CI: 68.2–81.5%). Despite this trade-off, the model maintained a strong F1-score of 83.3% (95% CI: 78.4–87.8%).

Fibroids displayed a distinct diagnostic profile characterized by high specificity (96.8%, 95% CI: 94.4–99.1%) and an accuracy of 90% (95% CI: 86.3–93.7%). In contrast, sensitivity was substantially lower at 47.1% (95% CI: 30.3–63.8%), with the model recognizing 16 TP and 18 FN cases. The precision for this class was 69.6% (95% CI: 50.8–88.4%), which, combined with the lower sensitivity, resulted in an F1-score of 56.1% (95% CI: 36.7–70.8%).

Analysis of the multiclass confusion matrix revealed distinct class-specific misclassification patterns (Table S2A,B). Of the 34 fibroid images, 16 were correctly classified, whereas 18 were misclassified as hyperplastic polyps. Functional polyps were correctly identified in 64 of 87 cases, while 23 were misclassified as hyperplastic polyps. Hyperplastic polyps showed the highest number of correct classifications, with 122 of 130 cases correctly identified. The remaining errors involved seven hyperplastic polyps classified as fibroids and one classified as a functional polyp. The predominant misclassification pattern was therefore the assignment of fibroids to the hyperplastic-polyp category, which contributed substantially to the low sensitivity observed for the fibroid class.

#### 3.2.2. Receiver Operating Characteristic (ROC) and Precision-Recall (PR) Analysis

To evaluate the discriminative capacity of the model across all possible decision thresholds independently of class imbalance, Receiver Operating Characteristic (ROC) analysis was performed.

**Figure 2:**
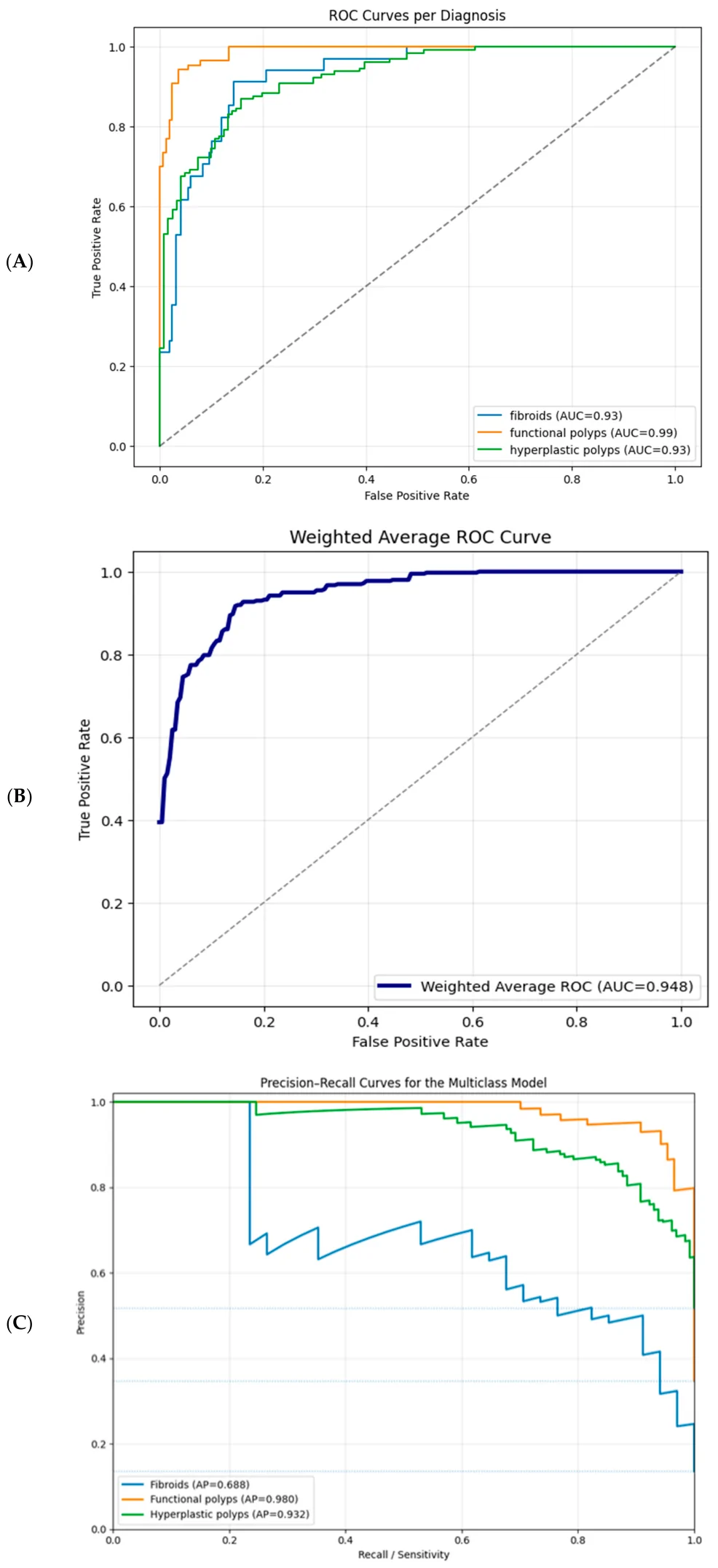
Receiver Operating Characteristic (ROC) and Precision-Recall (PR) analysis of the multiclass prediction model. (**A**) Class-specific ROC curves. (**B**) Weighted-average ROC curve. (**C**) Class-specific PR curves with the corresponding Average Precision (AP) values.

As illustrated in the class-specific ROC plot (Figure 2A), the network demonstrated high discriminatory power for all pathologies. The Area Under the Curve (AUC) reached 98.9% (95% CI: 98–99.7%) for functional polyps, followed by equally robust performances of 92.7% (95% CI: 88.8–96.7%) for submucosal fibroids and 92.5% (95% CI: 89.5–95.6%) for hyperplastic polyps.

Furthermore, to quantify the global discriminatory capability of the multiclass framework, class-specific and aggregate ROC analyses were performed. The weighted-average ROC curve is presented in Figure 2B and yielded an overall weighted-average AUC of 94.8% (95% CI: 92.5–96.9%). The corresponding macro-average and micro-average AUC values were 94.7% (95% CI: 92.2–96.7%) and 94.7% (95% CI: 92.5–96.5%), respectively (Table S3). The close agreement among the macro-, micro-, and weighted-average AUC estimates indicates consistent discriminatory performance across the three diagnostic categories.

Because ROC-AUC may provide an overly optimistic impression of performance in the presence of class imbalance, class-specific PR curves were additionally examined (Figure 2C). The AP was 68.8% (95% CI: 53.2–82.1%) for fibroids, 98.0% (95% CI: 96–99.3%) for functional polyps, and 93.2% (95% CI: 89.8–96.2%) for hyperplastic polyps. The weighted AP across the three diagnostic categories was 91.6% (95% CI: 88.3–93.4%). The lower AP observed for fibroids, compared with the two polyp categories, indicates a generally less favorable PR relationship across decision thresholds.

### 3.3. Training Convergence and Diagnostic Performance of the Binary Models

To optimize differential diagnosis between specific pathologies, three independent pairwise binary classification models were developed and evaluated.

#### Pairwise Convergence Analysis

**Figure 3:**
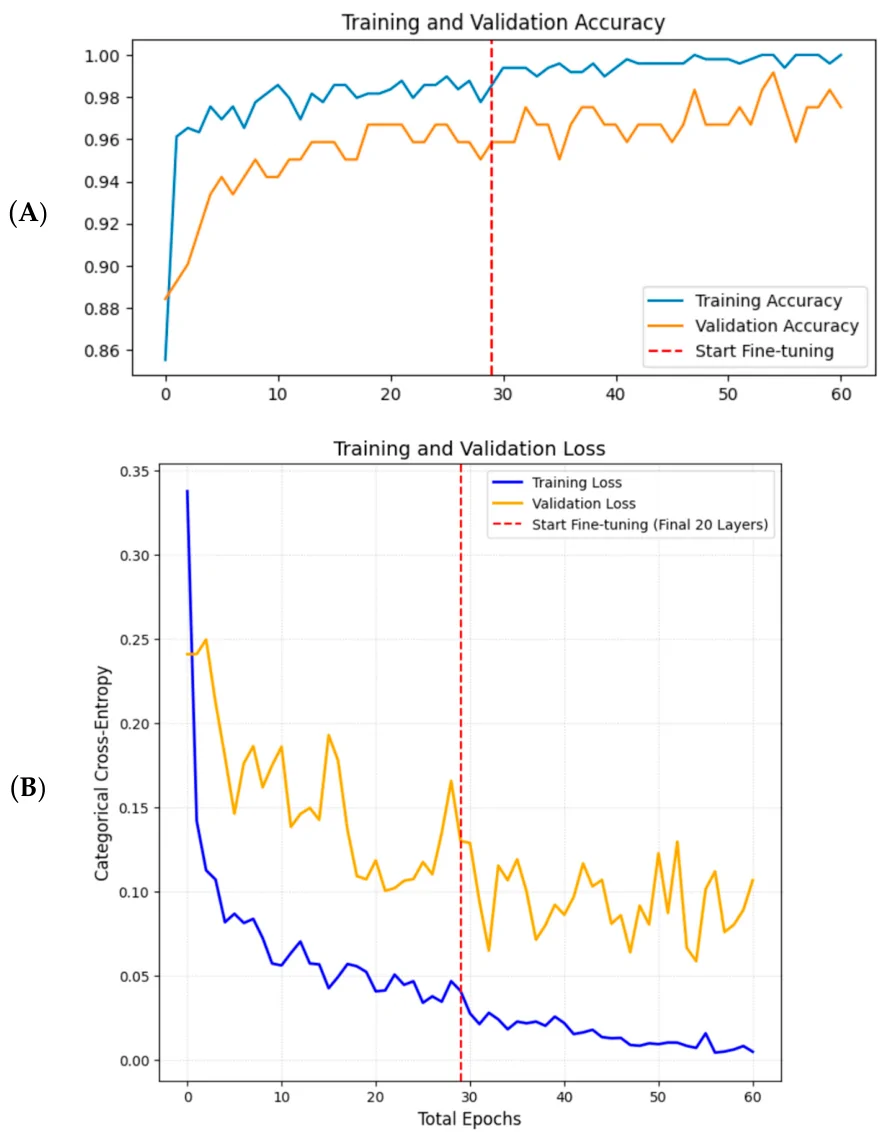

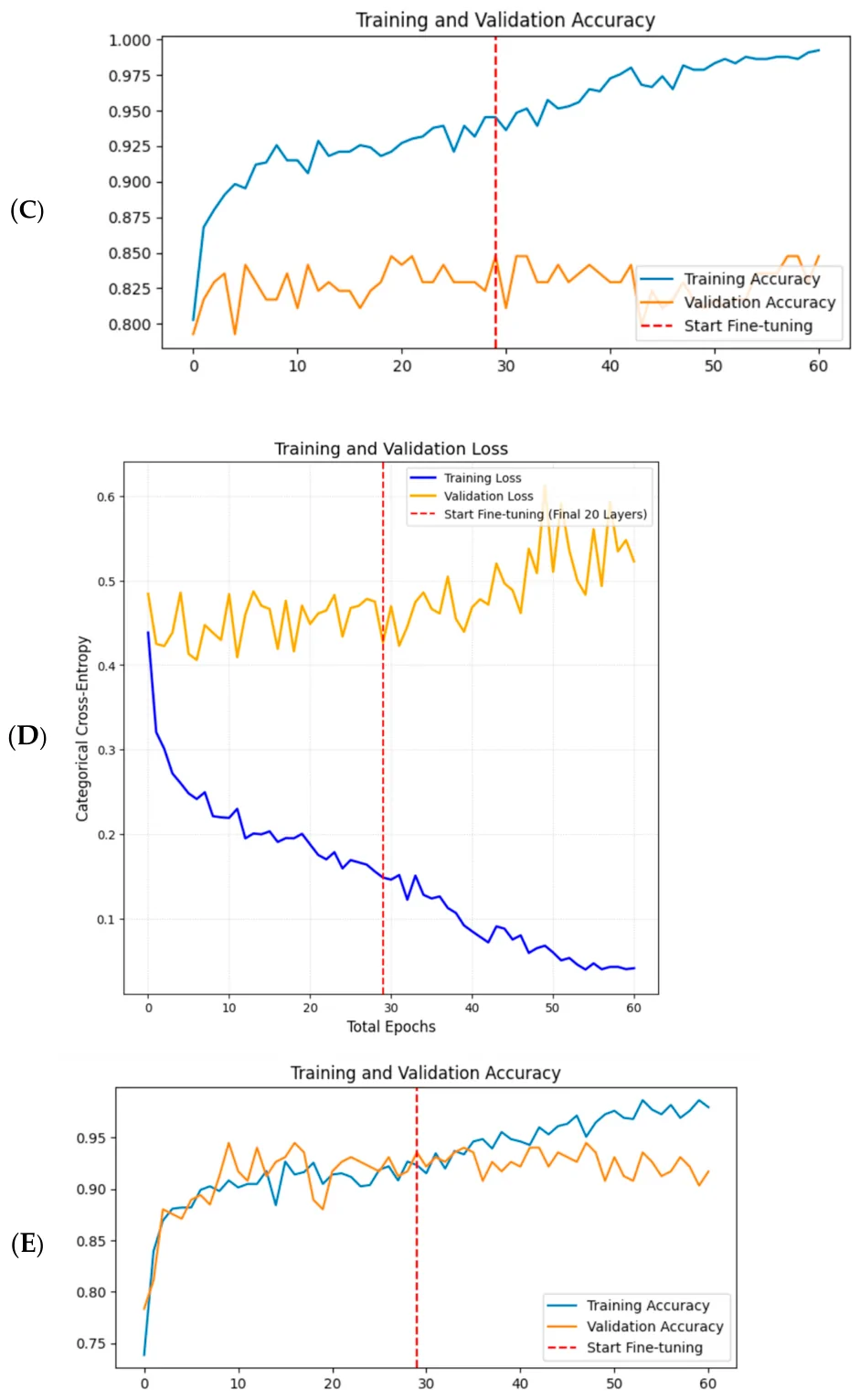

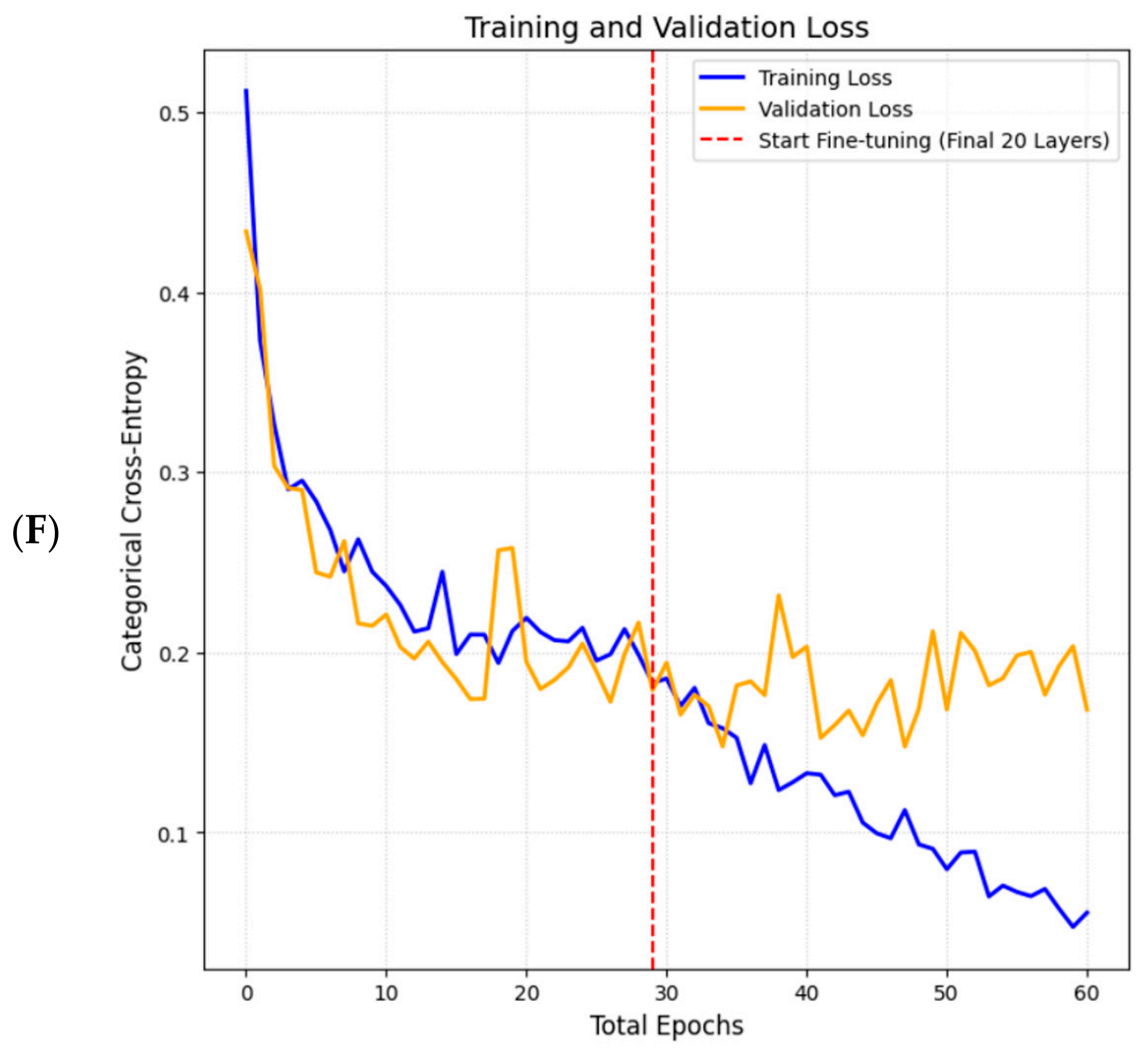
Dual-phase learning curves for binary models. (**A**) Training and validation accuracy for binary model 1. (**B**) Training and validation cross-entropy loss for binary model 1. (**C**) Training and validation accuracy for binary model 2. (**D**) Training and validation cross-entropy loss for binary model 2. (**E**) Training and validation accuracy for binary model 3. (**F**) Training and validation cross-entropy loss for binary model 3.

Each binary classification model was trained for 60 epochs, with selective fine-tuning of the final 20 convolutional layers initiated at epoch 30 (Figure 3). The learning gap was calculated at each epoch as the difference between training and validation accuracy, while the loss gap was calculated as validation loss minus training loss (Figure S1B–D, Table S1).

Model 1 demonstrated the most stable convergence. Its best validation accuracy was 99.17% at epoch 54, with an accuracy gap of only 0.83 percentage points. The maximum gap was 6.88 percentage points, and the final gap decreased to 2.48 percentage points. At the final epoch, training and validation losses were 0.0044 and 0.1066, respectively, indicating limited overfitting and good generalization to the validation data.

Model 2 showed the most pronounced overfitting among the binary models. Its best validation accuracy was 84.76% at epoch 20, with an accuracy gap of 7.42 percentage points. The gap increased to a maximum of 18.12 percentage points at epoch 51 and remained high at 14.9 percentage points at the final epoch. The final training loss was 0.0381, compared with a validation loss of 0.5226, indicating substantial divergence between training performance and validation generalization.

Model 3 showed an intermediate pattern. Validation accuracy was highest at 94.47% at epoch 10, when validation accuracy temporarily exceeded training accuracy and the accuracy gap was −4.97 percentage points. However, the gap increased during fine-tuning, reaching a maximum of 8.09 percentage points at epoch 59 and ending at 6.98 percentage points. The final training and validation losses were 0.0451 and 0.1683, respectively, indicating moderate late-stage overfitting but better generalization than model 2.

### 3.4. Diagnostic Performance and ROC Analysis of Binary Models

**Figure 4:**
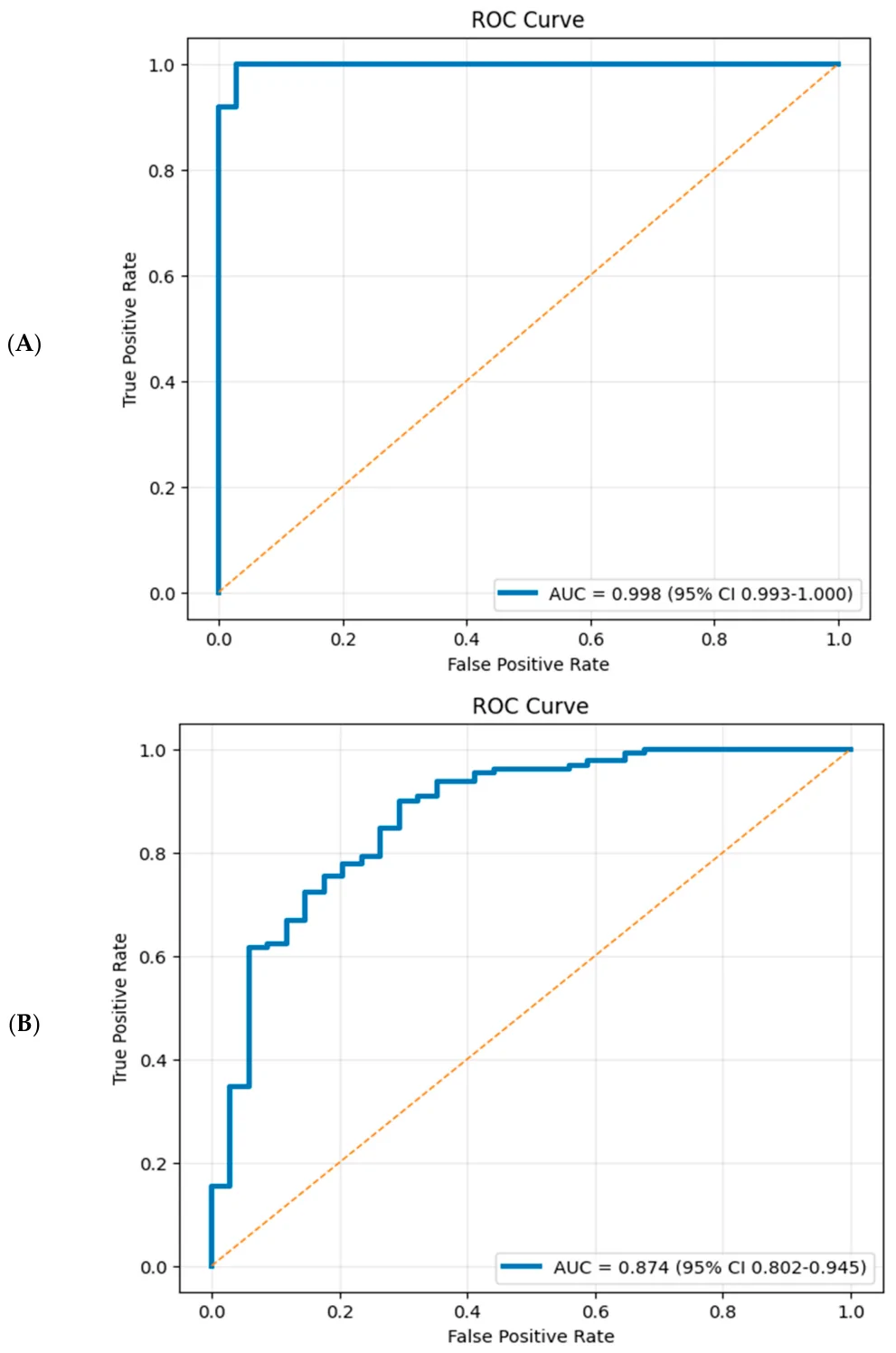

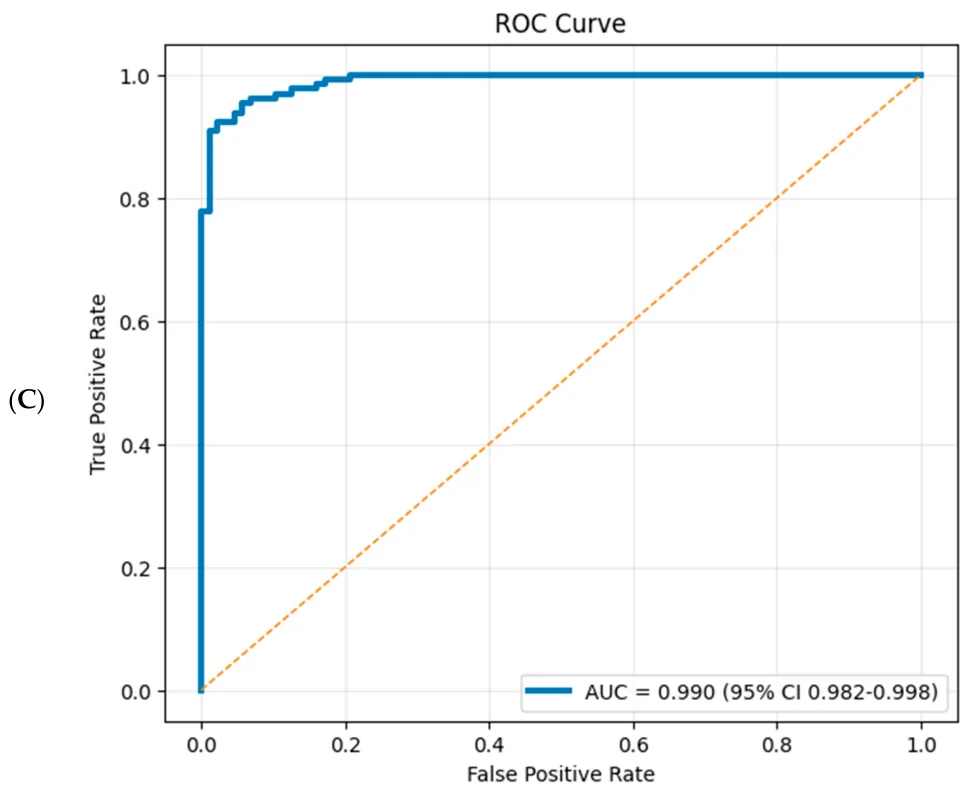
Receiver Operating Characteristic (ROC) curves of the binary classification models. (**A**) ROC curve of binary model 1. (**B**) ROC curve of binary model 2. (**C**) ROC curve of binary model 3.

The quantitative classification outcomes, including TP, TN, FP, and FN, together with the corresponding performance metrics are summarized in Table S4 and Table 2. The discriminatory performance of each binary classifier was further evaluated using ROC curve analysis, with the corresponding curves presented in Figure 4.

Model 1 achieved high overall diagnostic performance. The classifier achieved an accuracy of 96.7% (95% CI: 93.5–99.9%) and generated no FP predictions, resulting in perfect precision (100%) and specificity (100%). Sensitivity remained high at 88.2% (95% CI: 77.4–99.1%), corresponding to an F1-score of 93.8% (95% CI: 86.3–98.7%). The exceptional discriminative capability of this model was further confirmed by ROC analysis, which yielded a near-perfect AUC of 99.8% (95% CI: 99.3–100.0%).

Model 2 proved to be a challenging binary classification task. Despite achieving an overall accuracy of 87.2% (95% CI: 82.1–92.3%) and maintaining high specificity at 94.6% (95% CI: 90.7–98.5%), the model exhibited a substantially lower sensitivity of 58.8% (95% CI: 42.2–75.3%) due to 14 FN classifications. Consequently, the F1-score was limited to 65.6% (95% CI: 49.1–78.8%). These findings were mirrored by the ROC analysis, which produced an AUC of 87.4% (95% CI: 80.2–94.5%).

Finally, model 3 exhibited highly effective discrimination between the two polyp subtypes. The model achieved an overall accuracy of 92.2% (95% CI: 88.6–95.7%), a precision of 97.3% (95% CI: 93.6–100%) and a specificity of 98.4% (95% CI: 96.4–100%). Sensitivity reached 82.8% (95% CI: 74.8–90.7%), resulting in a strong F1-score of 89.4% (95% CI: 83.6–94.0%). ROC curve analysis further confirmed the model’s outstanding discriminatory capacity, with an AUC of 99.0% (95% CI: 98.2–99.8%).

### 3.5. Comparison with the EfficientNetV2 Benchmark

The comparative performance results for the ConvNeXt-XLarge and EfficientNetV2 architectures are summarized in Table 3, whereas the complete performance metrics and corresponding 95% CΙs for the EfficientNetV2 benchmark are presented in Table S5.

For the multiclass task, ConvNeXt-XLarge achieved a significantly higher accuracy than EfficientNetV2, with an 8.4 percentage-point difference (p=0.01). The difference in weighted multiclass AUC was also statistically significant, favoring ConvNeXt-XLarge by 8.3 percentage points (p=<0.001). For model 1, ConvNeXt-XLarge showed numerically higher accuracy and AUC than EfficientNetV2; however, neither difference reached statistical significance. The accuracy difference was 3.3 percentage points (p=0.22), while the AUC difference was 1.8 percentage points (p=0.67). For model 2, ConvNeXt-XLarge demonstrated a statistically significant accuracy advantage of 14.6 percentage points (p=<0.001). Nevertheless, the corresponding AUC difference of 4.7 percentage points was not statistically significant (p=0.48). For model 3, the observed differences in accuracy and AUC were 3.2 and 2.6 percentage points, respectively, and neither reached statistical significance (p=0.14 and p=0.33, respectively).

Overall, ConvNeXt-XLarge demonstrated statistically significant superiority over EfficientNetV2 for both accuracy and weighted AUC in the multiclass task, as well as for accuracy in model 2. Although ConvNeXt-XLarge showed numerically higher performance across all evaluated tasks, the differences for models 1 and 3, and the AUC difference for model 2, were not statistically significant.

### 3.6. Effect of Fine-Tuning on ConvNeXt-XLarge Performance

ConvNeXt-XLarge models trained only with the initial feature-extraction phase were compared with their fine-tuned counterparts. The multiclass weighted-average ROC-AUC for the fine-tuned models exceeded that of the non-fine-tuned models by 0.51 percentage points (*p* = 0.6). The corresponding AUC differences for the three binary models, defined as fine-tuned minus non-fine-tuned, were −0.3% for model 1 (*p* = 0.97), 1.15% for model 2 (*p* = 0.65), and −0.01% for model 3 (*p* = 0.99), and none of these differences reached statistical significance (Table S6).

### 3.7. Inference Latency and Throughput

The multiclass ConvNeXt-XLarge model achieved a mean inference latency of 129.30 milliseconds (ms) per image, with a median latency of 128.54 ms and a 95th-percentile latency of 133.48 ms. The corresponding throughput was 7.73 images per second. The three binary ConvNeXt-XLarge models demonstrated comparable computational performance, as summarized in Table 4.

### 3.8. Occlusion-Sensitivity Analysis Results

**Figure 5:**
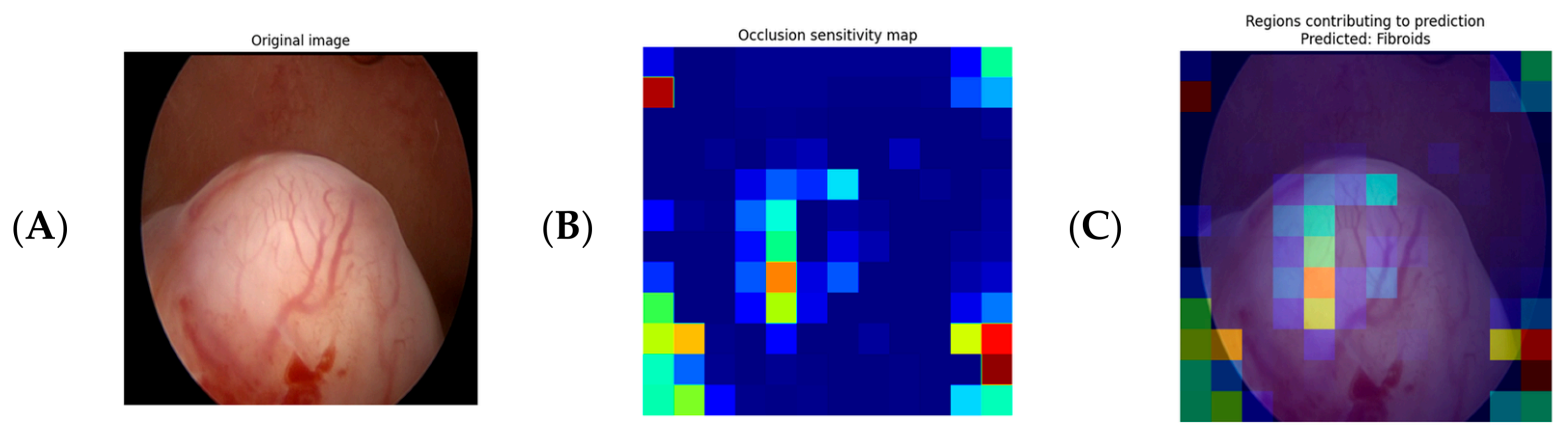
Representative occlusion-sensitivity analysis of a correctly classified fibroid. (**A**) Original hysteroscopic image. (**B**) Occlusion-sensitivity map. (**C**) Overlay of the sensitivity map on the original image.

Figure 5 presents a representative occlusion-sensitivity analysis for a correctly classified fibroid image. The ConvNeXt-XLarge model assigned the image to the fibroid class, consistent with the reference diagnosis. The strongest sensitivity was observed in a localized central region corresponding to the visually apparent fibroid. Lower-intensity responses were also observed in peripheral portions of the hysteroscopic field, which are consistently present as part of the image-acquisition geometry. In this example, the visualization suggests that the model relied predominantly on the central lesion-related region, while the peripheral responses should be interpreted as part of the overall visual context of hysteroscopic image formation.

## 4. Discussion

### 4.1. Primary Findings

The study’s main findings were as follows: (i) the ConvNeXt-XLarge multiclass prediction model achieved an overall weighted accuracy of 80.5%, a weighted AUC of 94.8%, and a weighted AP of 91.6% in classifying submucosal fibroids, functional polyps, and hyperplastic polyps; (ii) all three binary models demonstrated robust performance, with accuracies exceeding 87.2%; model 1 reaching an accuracy of 96.7% and AUC of 99.8%; (iii) the ConvNeXt-XLarge multiclass model performed significantly better than the corresponding EfficientNetV2 model, with 8.4% higher accuracy and 8.3% higher weighted AUC; (iv) among the binary ConvNeXt-XLarge models, only model 2 achieved significantly higher accuracy than the corresponding EfficientNetV2 model, with a 14.6% improvement.

### 4.2. Interpretation of Findings

Our ConvNeXt-XLarge multiclass prediction model achieved an overall accuracy of 80.5%. This outcome reflects the diagnostic challenge of distinguishing between these specific benign intrauterine lesions, which share highly overlapping morphological features. This performance closely mirrors the findings of Zhang et al. [32], who reported an accuracy of 80.8% using the VGGNet-16 architecture trained on 1851 images to differentiate among five pathologies, including endometrial polyps and submucosal leiomyomas. Similarly, Raimondo et al. [33] et al. trained the ResNet-50 architecture on 1500 images and achieved a slightly higher multiclass accuracy of 85.1%. However, their classification task involved the broader categories of focal benign lesions, diffuse benign lesions and premalignant or malignant lesions. In contrast, our multiclass model addressed the more challenging task of distinguishing among three benign focal lesions, specifically two polyp subtypes and submucosal fibroids.

When the diagnostic goal is shifted to binary classification, the discriminative power of the ConvNeXt-XLarge architecture becomes evident. Our binary models achieved high accuracies, reaching 96.7% in differentiating fibroids from functional polyps (model 1), 92.2% in distinguishing between the two polyp subtypes (model 3), and 87.2% in recognizing fibroids from hyperplastic polyps (model 2). Higher performance in binary settings is well documented in the literature. For instance, Zhang et al. [32] reported that simplifying a multiclass task into a binary classification of benign versus premalignant or malignant lesions increased model accuracy to 90.8%. Furthermore, in a recent study by Wang et al. [21], a ResNet-50 architecture trained on a large dataset of 49,646 images achieved 94.1% accuracy in distinguishing premalignant or malignant lesions from benign lesions.

### 4.3. Clinical Implications

The successful development of the ConvNeXt-XLarge multiclass and binary models carries important implications for diagnostic hysteroscopy. At present, hysteroscopic diagnosis remains highly operator-dependent and subjective, with performance varying substantially across clinicians and centers [3]. An AI-based framework that provides automated, high-accuracy classifications has the potential to act as an optical biopsy, offering a consistent second opinion that can help standardize lesion interpretation across operators with different levels of experience.

Practically, the model’s ability to differentiate between functional and hyperplastic polyps addresses a frequent intraoperative dilemma. Reliable distinction between benign, hormonally responsive endometrial changes and actively proliferating hyperplastic tissue may support more appropriate decisions regarding conservative management versus hysteroscopic resection. Likewise, accurate recognition of submucosal fibroids versus polyps may improve procedural planning, as these pathologies often require different resection techniques, instrumentation, and energy modalities. In this way, AI-assisted hysteroscopy could enhance diagnostic precision, optimize intraoperative decision-making and preoperative planning, and ultimately contribute to more consistent and potentially improved patient outcomes. Nonetheless, given the current single-center design, and evaluation on static images, the present system should be considered an adjunctive tool rather than a standalone diagnostic solution.

### 4.4. Strengths and Limitations

This study has several strengths. The primary strength lies in the deployment of the ConvNeXt-XLarge architecture, which successfully bridges the computational efficiency of CNNs with the global spatial awareness of ViTs, achieving high diagnostic accuracy. A selective-fine tuning strategy was employed, allowing task-specific adaptation to hysteroscopic features while preserving the robustness of the pretrained backbone and reducing the risk of overfitting. Furthermore, the study evaluated both a multiclass and three binary classification models, the latter being particularly useful for differential diagnosis between lesions with overlapping visual characteristics. In addition, the ConvNeXt-XLarge models were systematically compared with corresponding EfficientNetV2 baselines, enabling a rigorous performance assessment. Finally, occlusion-sensitivity mapping indicated that predictions were driven primarily by lesion-related image regions, supporting the clinical credibility of the model.

Despite these methodological advantages, several limitations should be acknowledged. First, the dataset was imbalanced across classes, with substantially fewer images of fibroids than polyps, which may have contributed to the lower sensitivity observed for fibroids in the multiclass model. Second, the study relied on an internal 80 to 20% train-validation split of data from a single center, without independent external validation. Consequently, the model’s generalizability across different clinical settings, patient populations, and imaging conditions have yet to be established. Third, the model was designed to classify only a limited set of intrauterine pathologies, specifically submucosal fibroids, functional polyps, and hyperplastic polyps, and did not account for the full spectrum of intracavitary lesions encountered in clinical practice. Furthermore, the model was developed and evaluated on static hysteroscopic images, which do not fully replicate the dynamic challenges of a live procedure, such as fluctuating illumination, the presence of blood, and rapid camera motion. Transitioning this model to real-time video analysis will be a vital next step in addressing these environmental variables. Finally, the learning-gap analysis revealed task-dependent overfitting during late fine-tuning, particularly in the multiclass model and model 2. The final training-validation accuracy gaps were 18.21 and 14.9 percentage points, respectively, indicating limited validation generalization despite selective fine-tuning.

### 4.5. Future Directions

Beyond the scope of the present work, the broader research landscape in AI-assisted hysteroscopy is likely to move towards real-time systems that operate directly on live endoscopic video streams, providing continuous lesion detection, classification, and segmentation throughout the procedure. In parallel, prospective studies that link AI-based optical diagnosis to management decisions and patient outcomes will be essential to determine how such technologies can be safely and effectively integrated into everyday clinical practice. Finally, future research may also explore combining hysteroscopic AI outputs with other clinical information to build multimodal decision-support tools that offer more comprehensive and personalized assessments than image-based models alone. In this context, recent work in wearable robotics has shown that integrating multiple physiological and biomechanical signals within a DL framework can enhance the robustness and reliability of AI-driven assistance, supporting the rationale for multimodal integration in clinical decision support as well [34].

## 5. Conclusions

This study demonstrates the potential of AI, specifically the ConvNeXt-XLarge architecture, to automate the classification of common uterine pathologies from high-resolution hysteroscopic images. The developed models demonstrated strong performance in differentiating between submucosal leiomyomas, functional polyps, and hyperplastic polyps. The primary multiclass model achieved an overall weighted accuracy of 80.5%, a weighted AUC of 94.8%, and a weighted AP of 91.6%. Notably, performance was higher in the binary classification tasks, with accuracy reaching 96.7% when distinguishing between leiomyomas and functional polyps, accompanied by an AUC of 99.8%. Importantly, systematic comparison with EfficientNetV2 benchmarks showed that ConvNeXt-XLarge provided superior performance in the multiclass task in terms of both accuracy and AUC, and in model 2 in terms of accuracy, suggesting that the deeper ConvNeXt-XLarge architecture may offer a meaningful advantage over a lighter CNN backbone for this challenging hysteroscopic classification task.

## Figures and Tables

**Table 1:** Evaluation metrics of multiclass prediction model.

	Accuracy % (95% CIs)	Precision % (95% CIs)	Recall/Sensitivity % (95% CIs)	Specificity % (95% CIs)	F1-Score % (95% CIs)	AUC % (95% CIs)	AP (95% CIs)
Fibroids	90 (86.3, 93.7)	69.6 (50.8, 88.4)	47.1 (30.3, 63.8)	96.8 (94.4, 99.1)	56.1 (36.7, 70.8)	92.7 (88.8, 96.7)	68.8 (53.2, 82.1)
Functional polyps	90.4 (86.8, 94.1)	98.5 (95.5, 100)	73.6 (64.3, 82.8)	99.4 (98.2, 100)	84.2 (77, 89.7)	98.9 (98, 99.7)	98 (96, 99.3)
Hyperplastic polyps	80.5 (75.6, 85.4)	74.9 (68.2, 81.5)	93.9 (89.7, 98)	66.1 (57.7, 74.6)	83.3 (78.4, 87.8)	92.5 (89.5, 95.6)	93.2 (89.8, 96.2)
Overall (weighted)	80.5 (75.6, 85.4)	82.3 (77.6, 87)	80.5 (75.6, 85.4)	81.8 (77, 86.6)	79.9 (74.7, 84.8)	94.8 (92.5, 96.9)	91.6 (88.3, 93.4)

CI: confidence intervals; AUC: area under the curve; AP: average precision.

**Table 2:** Evaluation metrics of binary prediction models.

	Accuracy % (95% CIs)	Precision % (95% CIs)	Recall/Sensitivity % (95% CIs)	Specificity % (95% CIs)	F1-Score % (95% CIs)	AUC % (95% CIs)
Model 1	96.7 (93.5, 99.9)	100 (100, 100)	88.2 (77.4, 99.1)	100 (100, 100)	93.8 (86.3, 98.7)	99.8 (99.3, 100)
Model 2	87.2 (82.1, 92.3)	74.1 (57.6, 90.6)	58.8 (42.2, 75.3)	94.6 (90.7, 98.5)	65.6 (49.1, 78.8)	87.4 (80.2, 94.5)
Model 3	92.2 (88.6, 95.7)	97.3 (93.6, 100)	82.8 (74.8, 90.7)	98.4 (96.4, 100)	89.4 (83.6, 94)	99 (98.2, 99.8)

Model 1: Fibroids vs. functional polyps binary model; Model 2: Fibroids vs. hyperplastic polyps binary model; Model 3: Functional polyps vs. hyperplastic polyps binary model; AUC: Area under the curve.

**Table 3:** Performance comparison between the ConvNeXt-XLarge and EfficientNetV2 architectures.

	Accuracy Difference %	*p*-Value	AUC Difference %	*p*-Value
Multiclass model (overall)	8.4	0.01	8.3	<0.001
Model 1	3.3%	0.22	1.8	0.67
Model 2	14.6	<0.001	4.7	0.48
Model 3	3.2	0.14	2.6	0.33

Model 1: Fiboids vs. functional polyps binary model; Model 2: Fibroids vs. hyperplastic polyps binary model; Model 3: Functional polyps vs. hyperplastic polyps binary model. Differences in accuracy and Receiver Operating Characteristic Area Under the Curve (ROC-AUC) were calculated as ConvNeXt-XLarge minus EfficientNetV2 and are reported as percentage-point differences. Accuracy differences were assessed using McNemar’s test for paired correct and incorrect predictions obtained from the same validation images. For models 1–3, AUC differences were assessed using the paired DeLong test for correlated ROC curves. The difference in the multiclass weighted AUC was assessed using a paired non-parametric permutation test, while its 95% confidence interval was estimated using paired bootstrap resampling. All tests were two-sided, and p < 0.05  was considered statistically significant.

**Table 4:** Computational benchmarking of the ConvNeXt-XLarge models.

Models	Mean Latency (ms/Image)	Median Latency (ms/Image)	SD (ms)	95 Percentile Latency (ms/Image)	Throughput (Images/s)
Multiclass model	129.30	128.54	2.03	133.48	7.73
Model 1	128.21	128.00	0.89	129.88	7.80
Model 2	127.29	127.30	0.65	128.18	7.86
Model 3	128.45	127.56	2.80	133.31	7.79

Model 1: Fiboids vs. functional polyps binary model; Model 2: Fibroids vs. hyperplastic polyps binary model; Model 3: Functional polyps vs. hyperplastic polyps binary model; ms: milliseconds; SD: Standard deviation; Throughput: Number of images processed per second.

## Data Availability

The data presented in this study are available on reasonable request from the corresponding author. The data is not publicly available due to privacy restrictions.

## Supplementary Materials

The following supporting information can be downloaded at: https://www.mdpi.com/article/10.3390/biology15171490/s1, Table S1: Learning-gap analysis at key training epochs for the multiclass and binary classification models; Table S2A: Multiclass confusion matrix of the ConvNeXt-XLarge model; Table S2B: Class-specific one-vs-rest classification counts of the multiclass model; Table S3: Summary of macro-, micro-, and weighted-average ROC-AUC estimates for the multiclass ConvNeXt-XLarge classification model; Table S4: Confusion matrix of binary ConvNeXt-XLarge prediction models; Table S5: Performance metrics of benchmark model EfficientNetV2; Table S6: AUC comparison between the ConvNeXt-XLarge models after fine tuning and before fine tuning; Figure S1: Learning-gap analysis of the ConvNeXt-XLarge models. (A) Multiclass model, (B) Model 1, (C) Model 2, and (D) Model 3.

## Abbreviations

The following abbreviations are used in this manuscript:

FIGO	International Federation of Gynecology and Obstetrics
AI	Artificial intelligence
CNN	Convolutional Neural Networks
DL	Deep learning
ViTs	Vision Transformers
GELU	Gaussian Error Linear Unit
ReLU	Rectified Linear Unit
OvR	One-vs-rest
TP	True Positive
TN	True Negative
FP	False Positive
FN	False Negative
ROC	Receiver Operating Characteristic
AUC	Area Under the Curve
CIs	Confidence Intervals
AP	Average Precision
