# Supplementary material for: Automated Classification of Endometrial Pathologies Using Artificial Intelligence
Source: Biology (Basel). 2026 Sep 2;15(17):1490. doi: 10.3390/biology15171490 (PMC13564762; doi:10.3390/biology15171490)
Supplement: Supplementary file 1 [file biology-15-01490-s001.zip › biology-4435091-supplementary.pdf]

Supplementary Material

Table S1 Learning-gap analysis at key training epochs for the multiclass and binary classification models.

Model	Reference point	Epoch	Training accuracy (%)	Validation accuracy (%)	Accuracy gap (percentage points)	Training loss	Validation loss	Loss gap
Multiclass model	Best validation accuracy	75	96.40	85.26	11.14	0.1095	0.5177	0.4082
	Maximum accuracy gap	92	98.93	80.48	18.45	0.0376	0.7390	0.7014
	Final epoch	100	99.48	81.27	18.21	0.0296	0.7497	0.7201
Model 1	Best validation accuracy	54	100.00	99.17	0.83	0.0058	0.0585	0.0527
	Maximum accuracy gap	2	96.14	89.26	6.88	0.1406	0.2409	0.1003
	Final epoch	60	100.00	97.52	2.48	0.0044	0.1066	0.1022
Model 2	Best validation accuracy	20	92.18	84.76	7.42	0.1911	0.4703	0.2792
	Maximum accuracy gap	51	99.22	81.10	18.12	0.0363	0.5915	0.5552
	Final epoch	60	99.66	84.76	14.90	0.0381	0.5226	0.4845
Model 3	Best validation accuracy	10	89.50	94.47	−4.97	0.2721	0.2149	−0.0572
	Maximum accuracy gap	59	98.41	90.32	8.09	0.0573	0.2036	0.1463
	Final epoch	60	98.69	91.71	6.98	0.0451	0.1683	0.1232

Model 1: Fiboids vs. functional polyps binary model; Model 2: Fibroids vs. hyperplastic polyps binary model; Model 3: Functional polyps vs. hyperplastic polyps binary model.

Table S2A. Multiclass confusion matrix of the ConvNeXt-XLarge model

True class	Predicted fibroids	Predicted functional polyps	Predicted hyperplastic polyps
Fibroids	16	0	18
Functional polyps	0	64	23
Hyperplastic polyps	7	1	122

Table S2B Class-specific one-vs-rest classification counts of the multiclass model

	**True Positive**	**True Negative**	**False Positive**	**False Negative**
Fibroids	16	210	7	18
Functional polyps	64	163	1	23
Hyperplastic polyps	122	80	41	8
Overall	202	126	49	49

Table S3. Summary of macro-, micro-, and weighted-average ROC-AUC estimates for the multiclass ConvNeXt-XLarge classification model.

AUC type	**% (95% CIs)**
Micro-average	94.7 (92.5, 96.5)
Macro-average	94.7 (92.2, 96.7)
Weighted-average	94.8 (92.5, 96.9)

ROC-AUC: Area Under the Receiver Operating Characteristic Curve; 95% CIs: 95% confidence intervals; Macro-average AUC: The unweighted mean of the one-vs-rest ROC-AUC values across the three classes, giving equal importance to each class; Micro-average AUC: Calculated by pooling all one-vs-rest decisions across samples and classes before computing a single ROC-AUC value; Weighted-average AUC is the mean of the class-specific one-vs-rest ROC-AUC values weighted according to the number of samples in each class.

Table S4. Confusion matrix of binary ConvNeXt-XLarge prediction models.

	**True Positive**	**True Negative**	**False Postive**	**False Negative**
Model 1	30	87	0	4
Model 2	20	123	7	14
Model 3	72	128	2	15

Model 1: Fiboids vs. functional polyps binary model; Model 2: Fibroids vs. hyperplastic polyps binary model; Model 3: Functional polyps vs. hyperplastic polyps binary model.

Table S5. Performance metrics of benchmark model EfficientNetV2.

	**Accuracy % (95% CIs)**	**Precision % (95% CIs)**	**Recall/Sensitivity % (95% CIs)**	**Specificity % (95% CIs)**	**F1-Score % (95% CIs)**	**AUC % (95% CIs)**

Multiclass model (overall)	72.1 (66.6, 77.7)	73.4 (67.9, 78.8)	72.1 (66.6, 77.7)	79.5 (74.5, 84.5)	72.4 (67.1, 77.8)	86.5 (82.3, 89.9)
Binary model 1	93.4 (89, 97.8)	90.6 (80.5, 100)	85.3 (73.4, 97.2)	96.6 (92.7, 100)	87.9 (78, 95.2)	97.9 (95.7, 100)
Binary model 2	72.6 (65.7, 79.4)	40 (27.1, 53)	67.7 (48.6, 80.8)	74.6 (967.1, 82.1)	49.4 (35.3, 61.9)	82.6 (75.9, 89.4)
Binary model 3	88.9 (84.7, 93.1)	100 (100, 100)	72.4 (63, 81.8)	100 (100, 100)	84 (77.7, 90.1)	96.4 (93.9, 99)

Binary model 1: Fiboids vs. functional polyps binary model; Binary model 2: Fibroids vs. hyperplastic polyps binary model; Binary model 3: Functional polyps vs. hyperplastic polyps binary model.

Table S6. AUC comparison between the ConvNeXt-XLarge models after fine tuning and before fine tuning.

	**AUC difference %**	**p-value**
Multiclass model (overall)	0.51	0.6
Model 1	-00.3	0.97
Model 2	1.15	0.65
Model 3	-0.01	0.99

Model 1: Fiboids vs. functional polyps binary model; Model 2: Fibroids vs. hyperplastic polyps binary model; Model 3: Functional polyps vs. hyperplastic polyps binary model. Differences in Receiver Operating Characteristic Area Under the Curve (ROC-AUC) were calculated as ConvNeXt-XLarge after fine tuning minus ConvNeXt-XLarge before fine tuning and are reported as percentage-point differences. For models 1–3, AUC differences were assessed using the paired DeLong test for correlated ROC curves. The difference in the multiclass weighted-average AUC was assessed using a paired non-parametric permutation test, while its 95% confidence interval was estimated using paired bootstrap resampling. All tests were two-sided, and $p < 0.05$ was considered statistically significant.

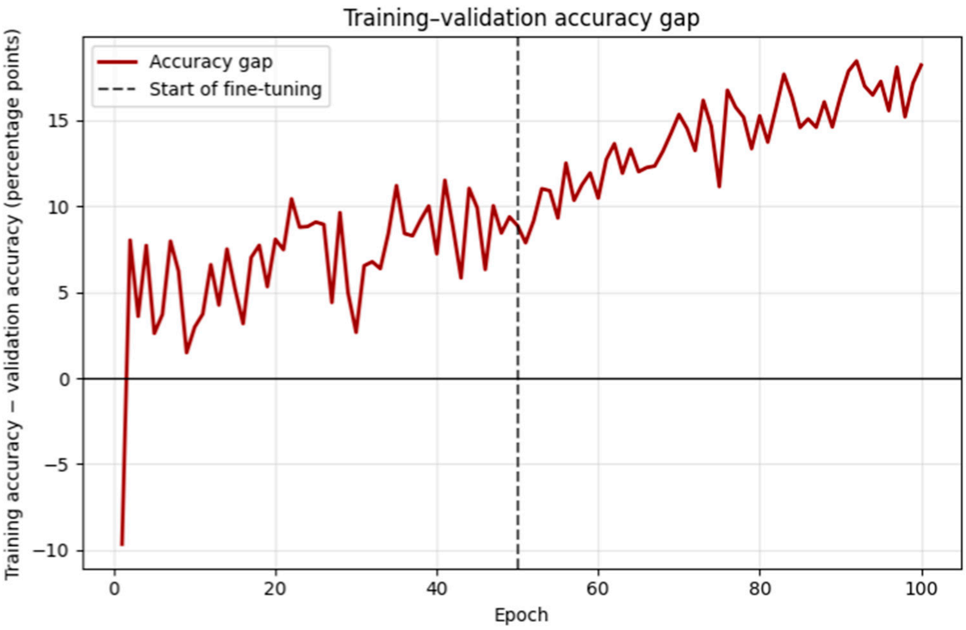
Training–validation accuracy gap
Accuracy gap
Start of fine-tuning
Training accuracy − validation accuracy (percentage points)
Epoch
15
10
5
0
−5
−10
0
20
40
60
80
100
A.

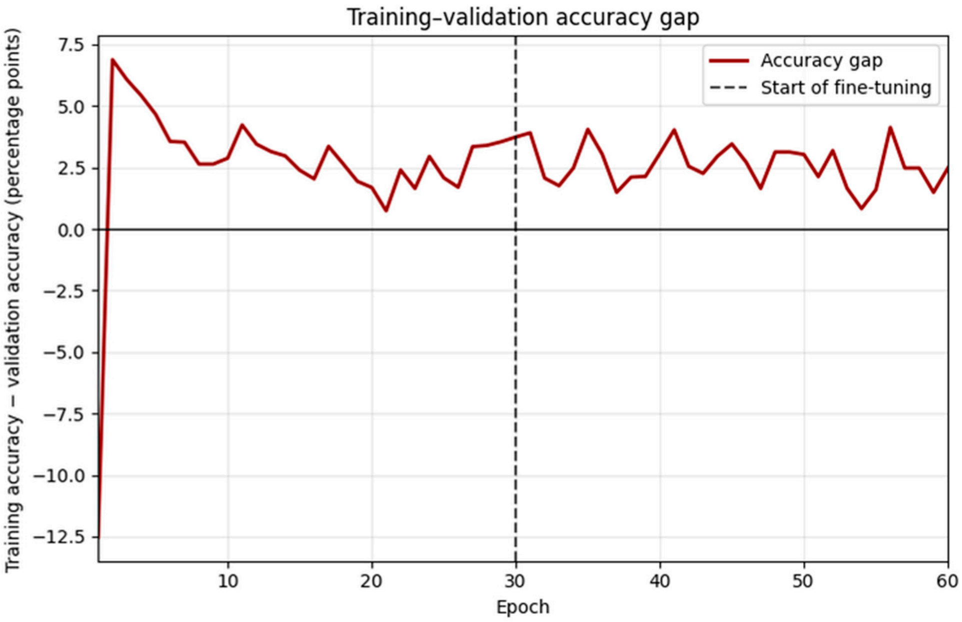
Training–validation accuracy gap
Accuracy gap
Start of fine-tuning
Training accuracy − validation accuracy (percentage points)
Epoch
7.5
5.0
2.5
0.0
−2.5
−5.0
−7.5
−10.0
−12.5
10
20
30
40
50
60
B.

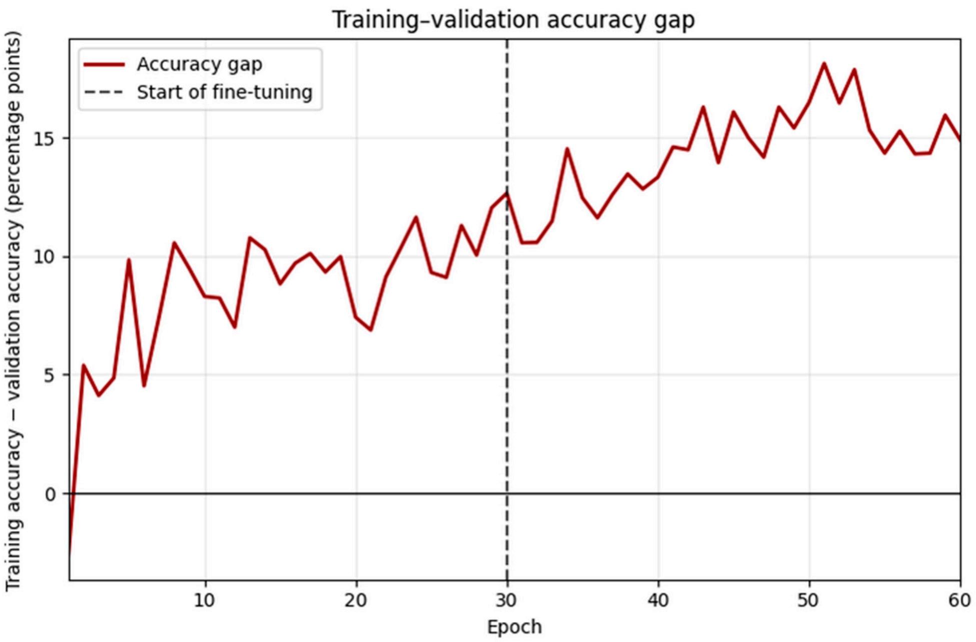

c.

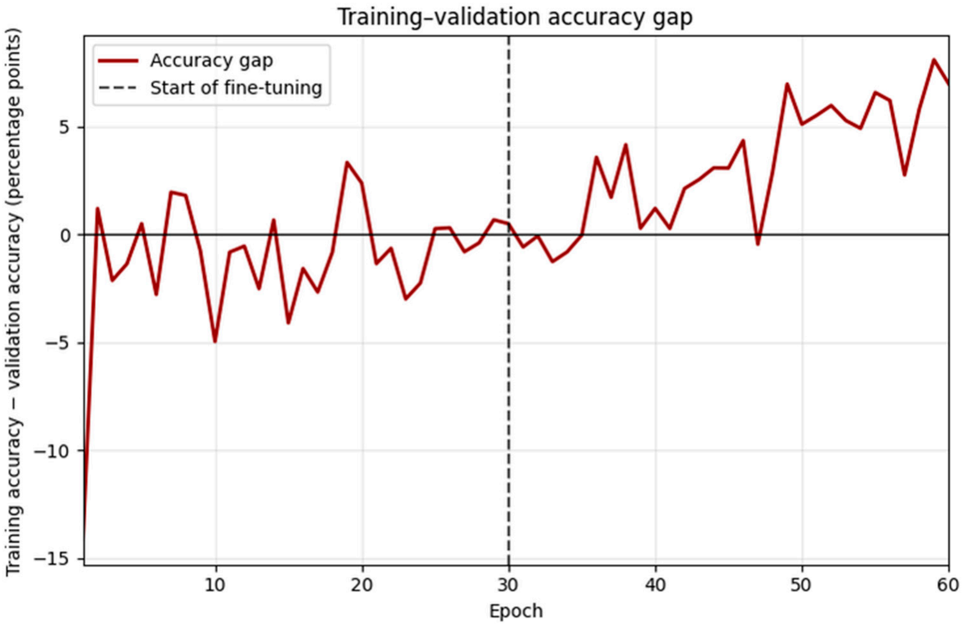

D.

Figure S1. Learning-gap analysis of the ConvNeXt-XLarge models. (A) Multiclass model. (B) Model 1. (C) Model 2. (D) Model 3.
